# dPRLR causes differences in immune responses between early and late feathering chickens after ALV-J infection

**DOI:** 10.1186/s13567-021-01016-7

**Published:** 2022-01-08

**Authors:** Guodong Mo, Bowen Hu, Qihong Zhang, Zhuohao Ruan, Wangyu Li, Jiaying Liang, Yizi Shen, Zhixin Mo, Zihao Zhang, Zhuyue Wu, Meiqing Shi, Xiquan Zhang

**Affiliations:** 1 Department of Animal Genetics, Breeding and Reproduction, College of Animal Science, South China Agricultural University, Guangzhou, 510642 Guangdong China; 2 Guangdong Provincial Key Lab of Agro-Animal Genomics and Molecular Breeding and Key Lab of Chicken Genetics, Breeding and Reproduction, Ministry of Agriculture and Rural Affairs, Guangzhou, 510642 Guangdong China; 3 Guangxi Key Laboratory of Livestock Genetic Improvement, Animal Husbandry Research Institute of Guangxi Zhuang Autonomous Region, Nanning, 530005 China; 4 Division of Immunology, Virginia-Maryland Regional College of Veterinary Medicine, University of Maryland, College Park, MD USA

**Keywords:** early feathering chickens, late feathering chickens, immune response, ALV-J, dPRLR

## Abstract

To understand the differences in immune responses between early feathering (EF) and late feathering (LF) chickens after infection with avian leukosis virus, subgroup J (ALV-J), we monitored the levels of prolactin, growth hormone and the immunoglobulins IgG and IgM in the serum of LF and EF chickens for 8 weeks. Moreover, we analysed the expression of immune-related genes in the spleen and the expression of *PRLR*, *SPEF2* and *dPRLR* in the immune organs and DF-1 cells by qRT–PCR. The results showed that ALV-J infection affected the expression of prolactin, growth hormone, IgG and IgM in the serum. Regardless of whether LF and EF chickens were infected with ALV-J, the serum levels of the two hormones and two immunoglobulins in EF chickens were higher than those in LF chickens (*P * < 0.05). However, the expression of immune-related genes in the spleen of positive LF chickens was higher than that in the spleen of positive EF chickens. In the four immune organs, *PRLR* and *SPEF2* expression was also higher in LF chickens than in EF chickens. Furthermore, the *dPRLR* expression of positive LF chickens was higher than that of negative LF chickens. After infection with ALV-J, the expression of *PRLR* in DF-1 cells significantly increased. In addition, overexpression of *PRLR* or *dPRLR* in DF-1 cells promoted replication of ALV-J. These results suggested that the susceptibility of LF chickens to ALV-J might be induced by *dPRLR*.

**Supplementary Information:**

The online version contains supplementary material available at 10.1186/s13567-021-01016-7.

## Introduction

Avian leukosis virus (ALV) is an oncogenic retrovirus that causes immunosuppression and neoplastic diseases [1]. According to the host range, the interference of viral envelopes and the pattern of cross-neutralization, ALVs can be divided into 10 subgroups (from A to J) [2]. ALV-J was first detected in the late 1980s and subsequently spread worldwide [3, 4]. To date, ALV-J has been found to infect many egg-type stocks and local Chinese breeds, resulting in significant economic losses in the poultry industry [4–7].

Sex identification of chicks can be based on the difference in rates of feather growth, which can be divided into an early feathering (EF) type and a late feathering (LF) type. Both the EF and LF types contain the prolactin receptor (*PRLR*) and sperm flagellar 2 (*SPEF2*) genes in their genetic structure. However, LF chickens have one more fusion gene, *dSPEF2/dPRLR*, and endogenous retroviruses 21 (*ev21*) compared with EF chickens [8–10]. Breeders use the different feather growth rates for sex determination and have found that EF and LF chickens respond differently to ALV-J infection. Some White Leghorn breeders have reported reduced production and slightly higher mortality in female progeny from dams carrying the *ev21* gene [11]. Breeders have had difficulties in eradicating ALV from most pure LF lines compared to EF chicken lines [12]. The existence of *ev21* increases the susceptibility of chickens to ALV infection and affects production performance and tumorigenesis [13–15].

However, a recent study revealed that some LF chickens lack the *ev21* gene and some EF chickens harbour the *ev21* gene [16]. Furthermore, *dPRLR* encodes a new prolactin (PRL) functional receptor that is widely expressed in all chicken tissues, and the pattern of spatiotemporal expression is likely to match that of the original *PRLR* gene. Importantly, PRLR and dPRLR were shown to be functionally coupled to the intracellular JAK/STAT signalling pathway in vitro [17]. PRL functions by first binding to its receptors and activating the JAK/STAT signalling pathway [18]. Growth hormone (GH) and PRL have similar structures and functions. Their receptors and signal transduction pathways are fundamentally the same [19]. After infection with ALV-J, it is not clear whether the levels of the two hormones PRL and GH and the two immunoglobulins IgG and IgM in the serum and the expression of some immune-related genes in the spleen differ between LF and EF chickens. On the other hand, both the *PRLR* and *dPRLR* genes are present in LF chickens. Whether these two genes have any effect on the immune responses of LF chickens infected with ALV-J remains unknown.

To understand the difference in immune responses between EF and LF chickens after infection with ALV-J, we detected the levels of PRL, GH, IgG, and IgM in the serum of positive and negative individuals of these two types of chickens for eight consecutive weeks. Furthermore, we analysed the expression of some immune-related genes in the spleen to illustrate the different immune responses of EF and LF chickens after infection with ALV-J.

## Materials and methods

### Ethics statement

All animal experiments in this study were conducted in accordance with protocols approved by the Institutional Animal Care and Use Committee of South China Agriculture University (No: SCAU 2018c008) and in accordance with the Animal Protection Law of the People’s Republic of China.

### Cells and antibodies

The chicken embryonic fibroblast cell line DF-1 was obtained from ATCC (Manassas, USA) and maintained in DMEM supplemented with 10% foetal bovine serum (FBS) and 0.1% penicillin/streptomycin at 37 °C in an atmosphere of 5% CO_2_. JE9, a specific mouse monoclonal antibody for the ALV-J envelope protein, was kindly provided by Prof. Aijian Qin (Yangzhou University). Goat anti-mouse IgG labelled with FITC was purchased from Bioss (China), while an ALV antigen-capture enzyme-linked immunosorbent assay kit was purchased from IDEXX (USA).

### Animals

LF and EF yellow chickens (140 days) were sourced from Chinese chicken farms in Guangdong Province, China. Virus isolation and identification were performed with DF-1 cells as we described previously [20]. The virus identification primer sequences are listed in Additional file 3. The virus identification and ALV-J viremia results are shown in Additional files 1, 4.

We used molecular identification methods to identify the feathering genotypes of samples. Using primers designed by Tixier-Boichard et al*.* [21], we amplified the *ev21* gene in our DNA samples. The expected PCR product of EF chickens was only a 515-bp band, while for LF chickens, there were two bands of 390 bp and 515 bp. We designed a pair of primers for *dSPEF2/dPRLR* gene amplification. A 1434-bp target fragment should be obtained in LF chickens but not in EF chickens. The primers are listed in Additional file 3. The feathering genotype detection results for LF and EF chickens are shown in Additional file 2.

Based on the results for virus isolation and identification and feathering genotype detection, 6 ALV-J-positive LF chickens, 6 ALV-J-negative LF chickens, 6 ALV-J-positive EF chickens and 6 ALV-J-negative EF chickens were selected. All animals were from the same farm. These selected chickens were raised separately, with a consistent feeding protocol among all individuals.

### Sample collection

Once each week for 8 weeks, we aseptically collected 2 mL of anticoagulated blood from each individual. The plasma was then separated by centrifugation at 2000 rpm and 4 ℃ for 15 min and stored at −80 ℃. All the samples were collected at the same time: 10:00 am every Monday of each week. Eight weeks later, the spleen, bone marrow, thymus, and caecal tonsils of each chicken were collected and stored at −80 ℃. The plasma samples were analysed with a p27 test for each collection, and the cell supernatant p27 test results are shown in Additional file 4.

### Determination of the levels of PRL, GH, IgG, and IgM in the serum by ELISA

The levels of PRL in the serum were measured using enzyme-linked immunosorbent assay (ELISA) kits for PRL (CLOUD-CLONE, Wuhan, China) following the manufacturer’s protocol. The levels of GH, IgG, and IgM were detected in the serum with specific ELISA kits purchased from Shanghai Enzyme-Linked Biotechnology Co., Ltd., (Shanghai, China) following the manufacturer’s protocol.

### Cell infection and gene overexpression

DF-1 cells were infected with ALV-J as previously described [22]. After an incubation for 24 h or 48 h in culture, we collected cells and extracted RNA and then measured *PRLR* and *SPEF2* expression by qRT–PCR. The laboratory ALV-J strain SCAU-HN06 was kindly provided by Prof. Weisheng Cao (South China Agricultural University, Guangzhou, China).

According to the *PRLR* sequence (NM_204854.1) in the NCBI database, Wuhan Genecreate Industrial Co., Ltd., was commissioned to construct pcDNA3.1-*PRLR* and pcDNA3.1-*dPRLR* plasmids. Then, we followed the method described by Li et al*.* [22] for cell transfection and infection. After an incubation for 24 h or 48 h in culture, we collected cells, extracted RNA, and then measured ALV-J viral gene expression by quantitative real-time polymerase chain reaction (qRT–PCR).

### RNA isolation and cDNA synthesis

Total RNA was extracted from tissues with RNAiso reagent (Takara, Japan) according to the manufacturer’s protocol. The integrity and quantity of RNA were assessed using 1% agarose gel electrophoresis and spectrophotometry (ND-2000, USA), respectively. cDNA was synthesized using MonScript™ RTIII All-in-One Mix (with dsDNse) (Monad Co., Ltd., Guangzhou, China) following the manufacturer’s protocol. The synthesized cDNA was stored at −20 ℃ until subsequent analysis using qRT–PCR.

### Quantitative real-time PCR

We previously performed RNA-seq (PRJNA552417) analysis of spleens from EF and LF chickens infected with ALV-J and identified some differentially expressed genes. Immune-related differentially expressed genes including *TLR4*, *TLR7*, *MDA5*, *SOCS3*, *VIP*, *IL-10*, *IRF1*, *NFкB*, *TNFα*, and *IL-1β* were selected, and the expression of each gene was detected in the spleen of LF and EF chickens. MonAmp™ SYBR^®^ Green qPCR Mix (Monad Co., Ltd., Guangzhou, China) was used for qRT–PCR on an ABI 7500 Real-Time Detection instrument (Applied Biosystems, USA) according to the manufacturer’s protocol. Relative gene expression was measured by qRT–PCR for each sample, and the nuclear gene GAPDH was used as a control. The primers used for qRT–PCR are shown in Additional file 3.

### Western blotting

Western blotting (WB) was performed as previously described [23]. The antibodies and their dilutions used for WB were as follows: the anti-ALV-J envelope protein monoclonal antibody JE9 (kindly provided by Prof. Aijian Qin, Yangzhou University; 1:1000), a rabbit anti-beta-actin antibody (Bioss, China; 1:500) and a goat anti-rabbit IgG H&L/HRP antibody (Bioss, China; 1:500).

### Statistical analyses

Statistical comparisons were performed using GraphPad Prism 5 (GraphPad Software Inc., USA). Data are presented as the mean  ±  one standard error of the mean (SEM). The statistical analyses were performed using one-factor analysis of variance, and statistical significance is represented by *P* values. *P*  < 0.05 was considered statistically significant, and *P* value bands of statistical significance are denoted as follows: *P  < 0.05, **P  <  0.01, and ***P  < 0.001.

## Results

### The serum PRL and GH levels of LF chickens are lower than those of EF chickens, regardless of infection status

**Figure 1:**
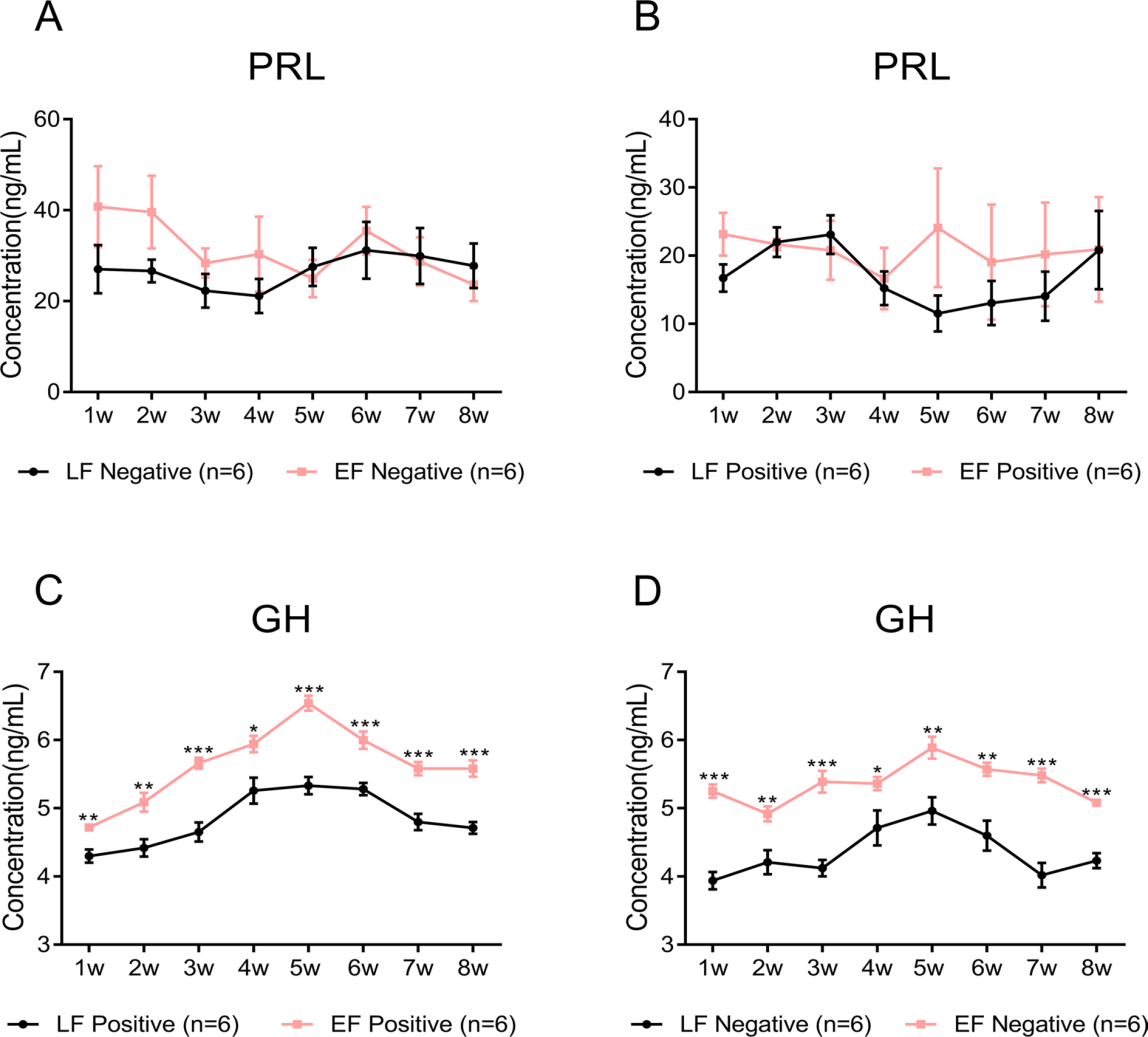
**The PRL and GH levels in the plasma of sampled chickens. A** PRL levels in the serum of negative LF and EF chickens. **B** PRL levels in the serum of positive LF and EF chickens. **C** GH levels in the serum of negative LF and EF chickens. **D** GH levels in the serum of positive LF and EF chickens. Positive LF, late feathering chickens infected with ALV-J; negative LF, late feathering chickens uninfected with ALV-J; positive EF early feathering chickens infected with ALV-J; negative EF early feathering chickens uninfected with ALV-J; n number of samples. The error bars represent one standard error of the mean (SEM) (*P  ≤ 0.05, **P  ≤ 0.01 and ***P  ≤ 0.001).

Comparing negative LF chickens with negative EF chickens, the PRL levels of the EF chickens were higher than those of the LF chickens except at the 5^th^, 7^th^ and 8^th^ weeks (Figure 1A). However, the PRL levels of positive EF chickens were higher than those of positive LF chickens in the 3rd week, while the 2nd and 8th weeks showed no difference, but were lower than those of positive LF chickens at the other times (Figure 1B). PRL and GH are similar in structure and function, and their receptors and signal transduction pathways are basically the same. Therefore, we also detected the serum GH content in LF and EF chickens. Intriguingly, regardless of the ALV-J infection status, the serum GH levels of LF chickens were always lower than those of EF chickens (Figures 1C, D). One explanation for these results is that PRL can bind to both PRLR and DPLR in LF chickens, which may lead to lower PRL levels in LF chickens than in EF chickens.

### The serum IgG and IgM levels of LF chickens are always lower than those of EF chickens

**Figure 2:**
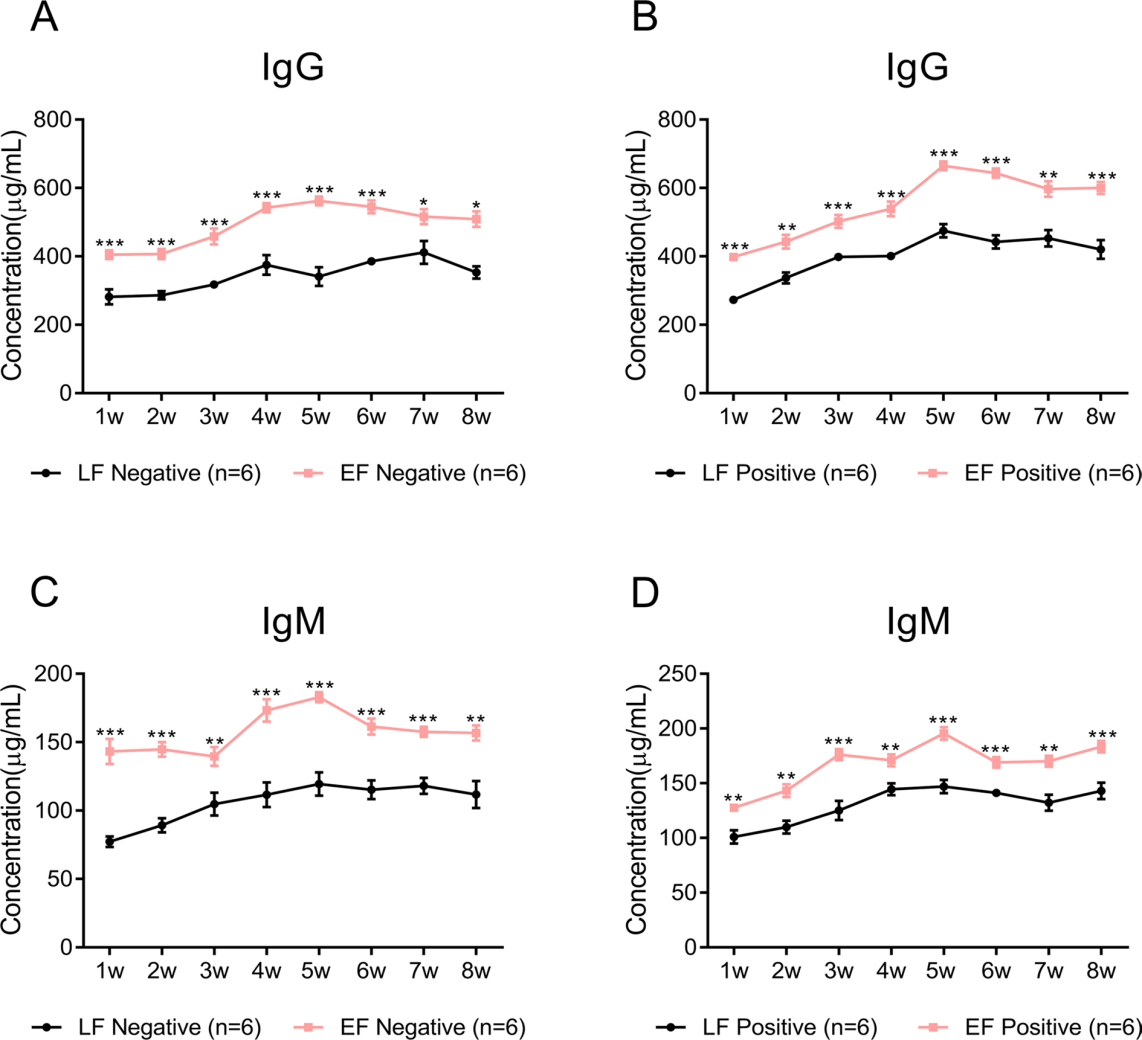
**IgG and IgM levels in the plasma of sampled chickens. A** IgG levels in the serum of negative LF and EF chickens. **B** IgG levels in the serum of positive LF and EF chickens. **C** IgM levels in the serum of negative LF and EF chickens. **D** IgM levels in the serum of positive LF and EF chickens.

Immunoglobulin refers to an animal protein with antibody activity. IgM is the first antibody isotype secreted in immune responses, while IgG is the most abundant antibody in the serum. Immunoglobulins play an important role in the function of the immune system. PRL can promote lymphocyte mitosis in a dose-dependent manner [24] and stimulate the proliferation of chicken splenocytes and thymocytes. Notably, regardless of whether chickens were infected with ALV-J, the IgG (Figures 2A, B) and IgM (Figures 2C, D) levels of LF chickens were always significantly lower than those of EF chickens. This might have been caused by the lower PRL levels in LF chickens compared to those in EF chickens, which might fail to increase the levels of IgM and IgG in the serum.

### The expression of most immune-related genes in the spleen of LF chickens is higher than that in the spleen of EF chickens

**Figure 3:**
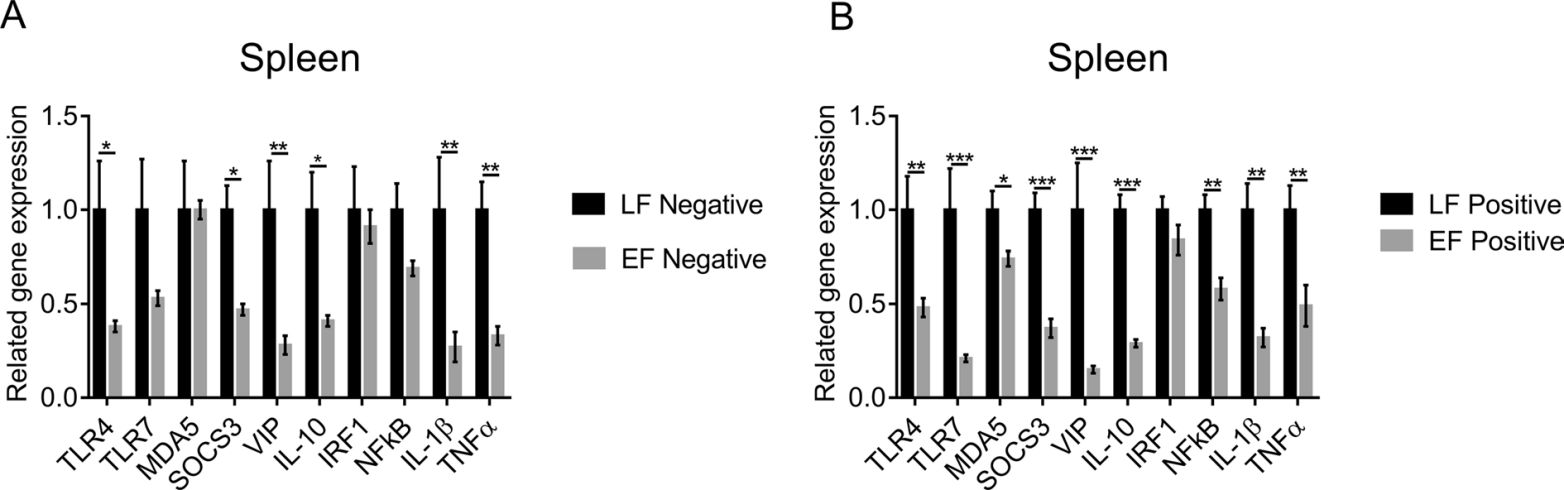
**The expression of immune-related genes in the spleen. A** Negative LF and EF chickens. **B** Positive LF and EF chickens.

In negative chickens, the expression of the *TLR4*, *TLR7*, *SOCS3*, *VIP*, *IL-10*, *IRF1*, *NFкB*, *TNFα*, and *IL-1β* genes but not that of the *MDA5* gene in EF chickens was lower than that in LF chickens. However, the differences in the expression of *TLR7*, *IRF1*, and *NFкB* between EF and LF chickens were not significant (*P*  > 0.05) (Figure 3A). In positive chickens, the expression of these genes other than *IRF1* in EF chickens was significantly lower than that in LF chickens (Figure 3B). This could be because PRL combines with PRLR and dPRLR to stimulate the expression of immune-related genes through the JAK/STAT signalling pathway. The LF chickens showed higher expression of most of the above immune-related genes because of the existence of *dPRLR*.

### The expression of *PRLR*, *SPEF2* and *dPRLR* in the immune organs of LF chickens is significantly higher than that in the immune organs of EF chickens

**Figure 4:**
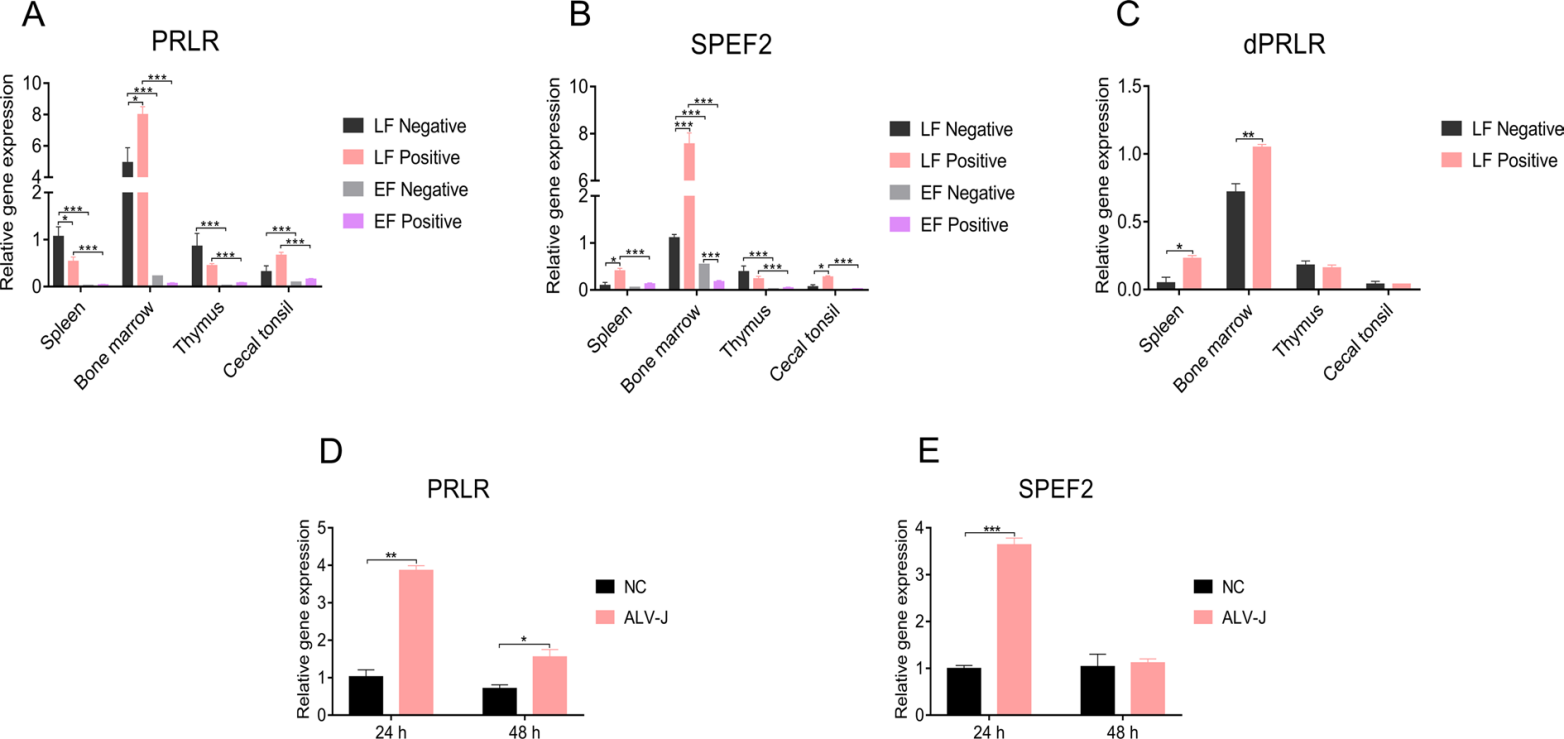
**Expression of the PRLR, SPEF2 and dPRLR genes in the four immune organs and DF-1 cells measured by qRT–PCR. A** The expression of *PRLR* in the four immune organs of LF and EF chickens. **B** The expression of *SPEF2* in the four immune organs of LF and EF chickens. **C** The expression of *dPRLR* in the four immune organs of LF chickens. **D** The expression of *PRLR* in DF-1 cells after infection with ALV-J. **E** The expression of *SPEF2* in DF-1 cells after infection with ALV-J.

We analysed the expression of the *PRLR*, *SPEF2* and *dPRLR* genes in the four immune organs, the spleen, bone marrow, thymus and caecal tonsils, by qRT–PCR. The results showed that the expression of the *PRLR* and *SPEF2* genes in each immune organ was significantly higher in LF chickens than in EF chickens (*P*  < 0.05). Compared with that in the immune organs of LF chickens, the *PRLR* and *SPEF2* expression in the four immune organs of EF chickens was very low (Figures 4A, B). In the spleen and thymus, the *PRLR* expression in negative LF individuals was higher than that in positive LF individuals. In the bone marrow and caecal tonsils, the *PRLR* expression in positive LF individuals was higher than that in negative LF individuals (Figure 4A). The *SPEF2* expression of positive LF individuals was higher than that of negative LF individuals for the spleen, bone marrow and caecal tonsils but not the thymus (Figure 4B). *dPRLR* expression in the spleen and bone marrow was higher in positive LF individuals than in negative LF individuals. However, there was no difference in *dPRLR* expression in the thymus or caecal tonsils between EF and LF chickens (Figure 4C).

After infection with ALV-J, *PRLR* and *SPEF2* expression in DF-1 cells was evaluated (Figure 4). The *PRLR* expression in the infection group was significantly higher than that in the control group at 24 h and 48 h (*P* < 0.05) (Figure 4D). The *SPEF2* expression in the infection group was significantly higher than that in the control group at 24 h (*P* < 0.01) (Figure 4E).

### Overexpression of *PRLR* or *dPRLR* promotes ALV-J replication

**Figure 5:**
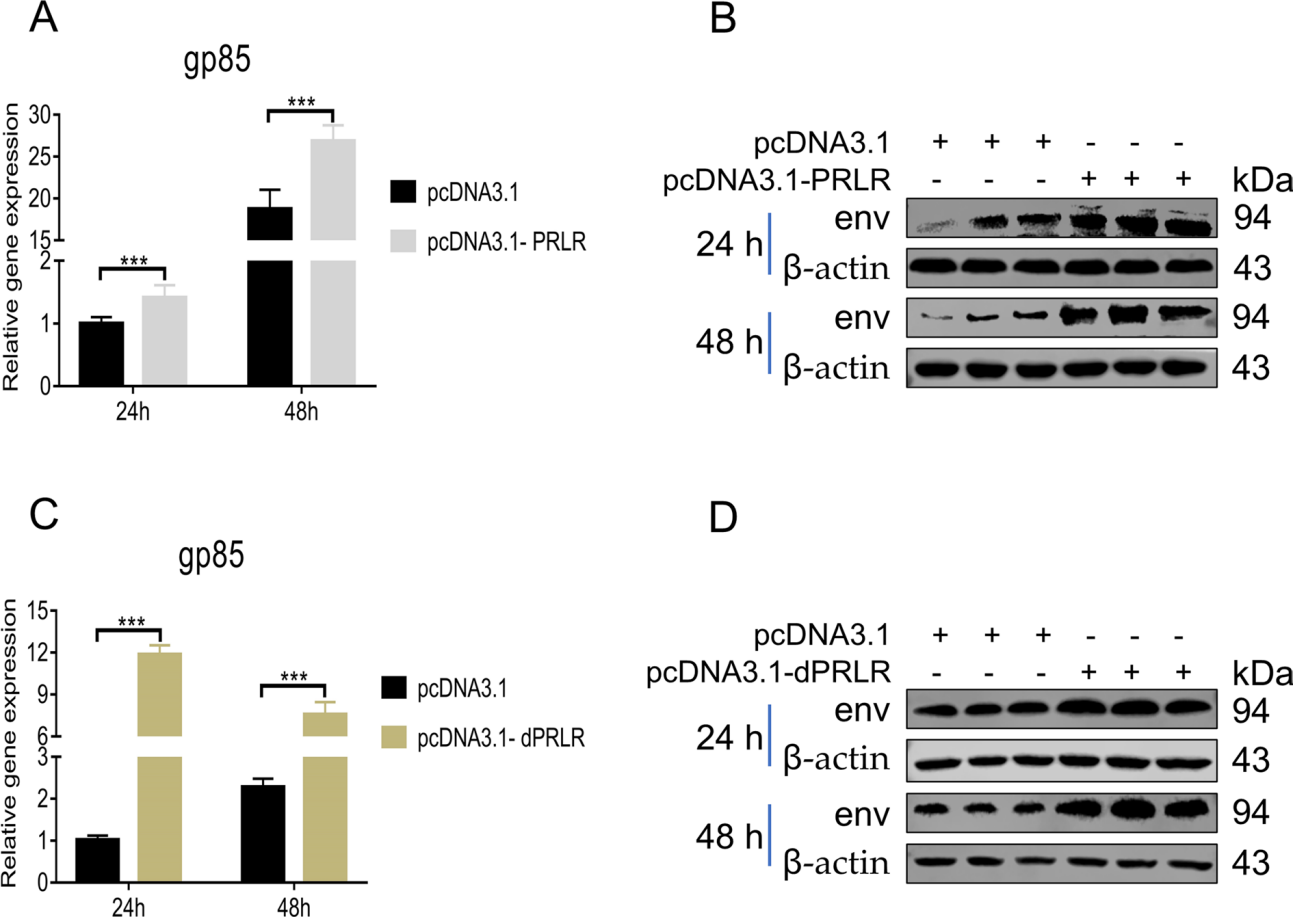
**Overexpression of PRLR or dPRLR promoted ALV-J replication.** The expression of the ALV-J *gp85* gene in *PRLR*-overexpressing DF-1 cells after infection with ALV-J measured by qRT–PCR (**A**) and WB (**B**). The expression of the ALV-J *gp85* gene in *dPRLR*-overexpressing DF-1 cells after infection with ALV-J measured by qRT–PCR (**C**) and WB (**D**).

Next, we overexpressed the *PRLR* and *dPRLR* genes separately in vitro to investigate the role of each gene in ALV-J replication in chickens. The results showed that overexpression of *PRLR* (Figures 5A, B) or *dPRLR* (Figures 5C, D) promoted the expression of the ALV-J *gp85* gene at 24 h and 48 h, indicating that *PRLR* and *dPRLR* significantly promoted ALV-J replication in chickens.

## Discussion

*Ev21* has been used as a molecular marker for LF detection in chickens. It is believed that the harbouring of *ev21* causes LF chicken susceptibility to ALV-J [13, 25, 26]. Beginning in the 1980s, researchers have reported reduced performance and slightly higher mortality in female progeny of dams carrying the *ev21* gene [11, 12]. Harris et al. [13] reported that decreased egg productivity was associated with an increased incidence of ALV-J. Fadly and Smith [27] concluded that the status of *ev21* may affect ALV-J infection and tumour development. Williams et al*.* [28] showed that harbouring *ev21* influenced ALV-J viremia and antibody production in some White Leghorn chickens. After inoculation with ALV-J at hatching, 5% of chickens lacking *ev21* were viremia tolerant compared with 54% of chickens harbouring *ev21* [28]*.* The incidence of tumours in chickens harbouring *ev21* (13.8%) was significantly higher (*P*  < 0.01) than that in chickens lacking *ev21* (2.6%) [29]. Collectively, these studies demonstrate that chickens harbouring *ev21* are more susceptible to ALV-J infection than are chickens lacking *ev21*.

In this study, we found that the levels of GH, IgG, and IgM in LF chickens (harbouring *ev21*) were significantly lower than those in EF chickens (lacking *ev21*), regardless of the ALV-J infection status. In LF chickens, the serum PRL levels were generally lower than those in EF chickens. The results further confirmed the difference in ALV-J infection between LF and EF chickens. However, the expression of most immune-related genes in the LF chicken spleen was higher than that in the EF chicken spleen. Therefore, we speculated that the *dPRLR* gene might regulate the immune response in LF chickens.

Some LF chickens lack the *ev21* gene, and some EF chickens harbour the *ev21* gene [16]. *dPRLR* is likely to encode a novel functional receptor for PRL and is widely expressed in all chicken tissues, and its spatiotemporal expression pattern is likely to match that of the *PRLR* gene [17]. Similar to the *PRLR* gene, *dPRLR* can significantly increase the level of STAT5 phosphorylation after activation [17]. In LF chickens, the serum PRL levels were generally lower than those in EF chickens, but the expression of most immune-related genes including *TLR4*, *TLR7*, *MDA5*, *SOCS3*, *VIP*, *IL-10*, *IRF1*, *NFкB*, *TNFα*, and *IL-1β* in the spleen was significantly higher in LF chickens than in EF chickens. PRL and GH are similar in structure and function, and their receptors and signal transduction pathways are basically the same. GH can also regulate the immune response through the JAK/STAT signalling pathway. When PRL levels decrease, the body may compensate by increasing GH levels. PRL can promote lymphocyte mitosis [24, 30]. Thus, the serum IgG and IgM levels of EF chickens were observed to be higher than those of LF chickens. This may be because there is one more *dPRLR* gene in LF chickens than in EF chickens.

We found that the expression of *PRLR* and *SPEF2* in the four immune organs in LF chickens was significantly higher than that in the corresponding immune organs in EF chickens. The expression of *dPRLR* in the spleen and bone marrow in positive LF individuals was higher than that in negative LF individuals, and the expression of *PRLR* in DF-1 cells was significantly increased after infection with ALV-J. Like *PRLR*, *dPRLR* significantly increases the level of STAT5 phosphorylation after activation [17]. The JAK/STAT signalling pathway is a signal transduction pathway stimulated by a variety of cytokines and is involved in multiple immune signalling pathways [31, 32]. These results indicate that PRLR and dPRLR play an important role in ALV-J infection. Okamura et al. [33] showed that the transcription of the *dSPEF2* gene possibly represses the expression of *dPRLR* mRNA and alters the alternative splicing bias in the 5′ UTR of *PRL* receptor mRNAs to increase translational efficiency. The immune functions of the SPEF2 and dSPEF2 genes are still unclear.

Receptors for growth factors, cytokines or hormones are clearly potential viral receptors, as they are already adapted to bind to specific circulating ligands and subsequently promote viral entry and survival by receptor-mediated endocytosis [34]. The receptors known to be used by viruses include insulin-like growth factor 1 receptor (IGF1R), platelet-derived growth factor receptor (PDGFR) and epidermal growth factor receptor (EGFR) [35, 36]. Recently, Griffiths et al. [37] found that IGF1R is an entry receptor for respiratory syncytial virus (RSV). RSV glycoprotein F binds to IGF1R, triggering activation of protein kinase C zeta, which recruits nucleolin from the nuclei of cells to the cell surface, where it probably facilitates RSV entry into the cell [37]. To infect a host cell, a virus must first bind to receptors on the host cell surface. PRLR and dPRLR clearly have potential as viral receptors. They are widely distributed in many tissues. Our present results demonstrated that overexpression of *PRLR* or *dPRLR* promoted the expression of the ALV-J *gp85* gene. Therefore, we speculated that *PRLR* and *dPRLR* are receptors for ALV-J. The presence of the *dPRLR* gene may be the reason why LF chickens are susceptible to ALV-J.

In conclusion, after infection with ALV-J, the levels of PRL, GH, IgG, and IgM in the serum and the expression of some immune-related genes in the spleen were different between LF and EF chickens. The reason why LF chickens are susceptible to ALV-J is probably due to the presence of the *dPRLR* gene.

## Supplementary Information

**Additional file 1. ALV-J isolation and identification**. **A** PCR detection results for the cell DNA of a positive sample using an ALV-J-specific primer. **B** PCR detection results for the cell DNA of a negative sample using an ALV-J-specific primer. **C** IFA results for DF1 cells using the ALV-J-specific antibody JE9 (200x magnification). bp base pairs. The numbers on the left indicate the lengths of molecular weight standards. M DL2000 marker; LF chickens L1-L12; EF chickens E1-E12; positive control +; negative control −; NC negative control. Note: When the supernatant p27 results for DF-1 cells incubated with the sample plasma were positive, the cell genome was amplified with the ALV-J-specific primer to obtain the target fragment (545 bp) (Additional file 1A). Other subgroups of ALV-, MDV- and REV-specific primers were used for amplification, and no relevant target fragments were obtained (data not shown). The target fragments were not obtained in the individuals with a negative result for the supernatant p27 test (Additional file 1B). To further confirm that the selected chickens were infected with the ALV-J subgroup, the positive samples were subjected to IFA verification. The plasma samples were used to infect DF-1 cells and showed obvious green fluorescence, indicating that the positive EF and LF chickens were infected with ALV-J, while the negative control group showed no green fluorescence (Additional file 1C). Furthermore, the plasma samples were analysed with a p27 test for each collection, and the cell supernatant p27 test results are shown in Additional file 4.

**Additional file 2. Detection of the *ev21*** **and** ***dSPEF2/dPRLR*** **genes in sampled chickens**. **A** The amplification results for the *ev21* gene. **B** The amplification results for the *dSPEF2/dPRLR* gene. M DL2000 marker; bp base pairs; LF late feathering chicken; 1-6 LF chickens infected with ALV-J; 7-12 LF chickens not infected with ALV-J; EF early feathering chicken; 1-6 EF chickens infected with ALV-J; 7-12 EF chickens not infected with ALV-J. Note: Two target fragments (515 and 390 bp) produced with *ev21* gene primers and a 1434-bp target fragment produced with *dSPEF2/dPRLR* gene primers were found for all LF chickens. Only one target fragment (515 bp) produced with *ev21* gene primers and no target fragment produced with *dSPEF2/dPRLR* gene primers were found for all EF chickens (Additional file 2A and B).

**Additional file 3. Primer information.**

**Additional file 4. ALV-J viremia was detected by ALV group-specific antigen (p27) ELISA**. Note: LF Po LF chickens infected with ALV-J; LF Ne LF chickens not infected with ALV-J; EF Po EF chickens infected with ALV-J; EF Ne EF chickens not infected with ALV-J; W, week. The results for viremia are expressed as the S/*P* value. An S/*P* value > 0.2 indicates the presence of ALV-J viremia.
