# Supplementary figures and images for: dPRLR causes differences in immune responses between early and late feathering chickens after ALV-J infection
Source: Vet Res. 2022 Jan 8;53:1. doi: 10.1186/s13567-021-01016-7 (PMC8742939; doi:10.1186/s13567-021-01016-7)

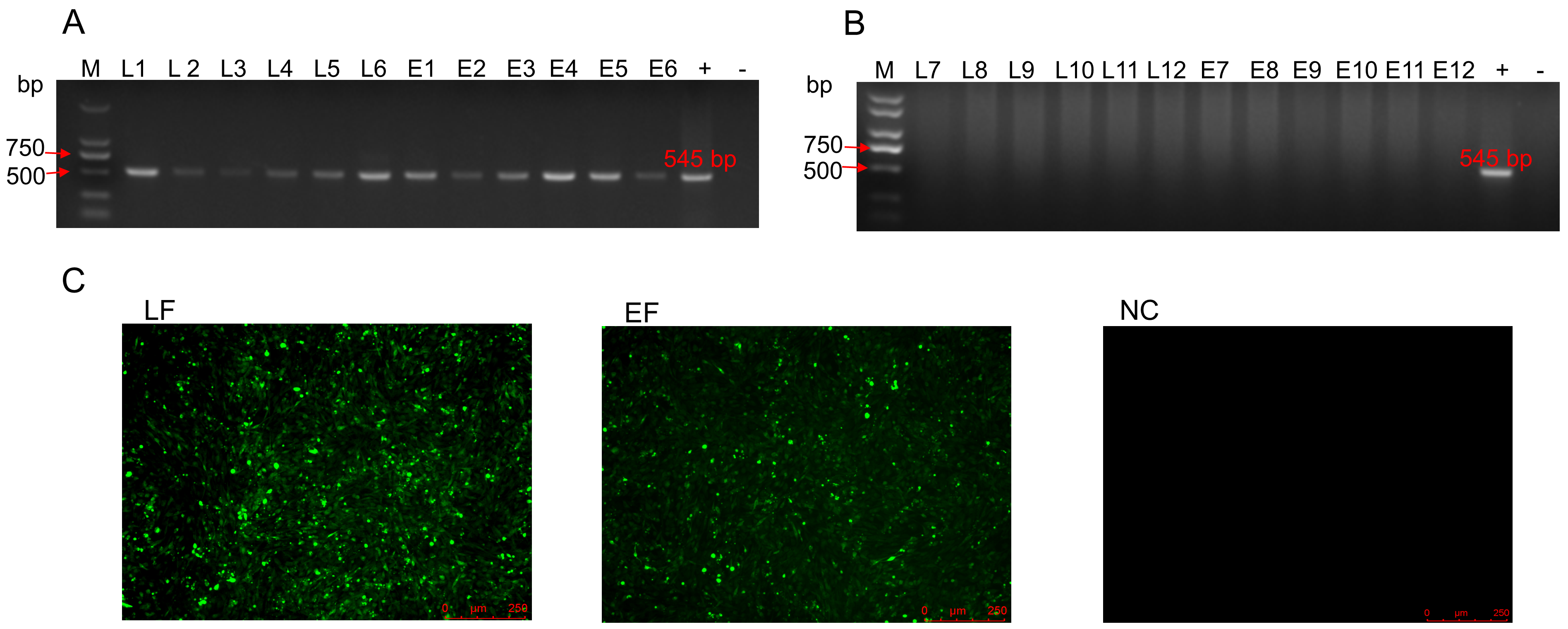

Supplement: Supplementary file 1 — Additional file 1. ALV-J isolation and identification. A PCR detection results for the cell DNA of a positive sample using an ALV-J-specific primer. B PCR detection results for the cell DNA of a negative sample using an ALV-J-specific primer. C IFA results for DF1 cells using the ALV-J-specific antibody JE9 (200x magnification). bp base pairs. The numbers on the left indicate the lengths of molecular weight standards. M DL2000 marker; LF chickens L1-L12; EF chickens E1-E12; positive control +; negative control −; NC negative control. Note: When the supernatant p27 results for DF-1 cells incubated with the sample plasma were positive, the cell genome was amplified with the ALV-J-specific primer to obtain the target fragment (545 bp) (Additional file 1A). Other subgroups of ALV-, MDV- and REV-specific primers were used for amplification, and no relevant target fragments were obtained (data not shown). The target fragments were not obtained in the individuals with a negative result for the supernatant p27 test (Additional file 1B). To further confirm that the selected chickens were infected with the ALV-J subgroup, the positive samples were subjected to IFA verification. The plasma samples were used to infect DF-1 cells and showed obvious green fluorescence, indicating that the positive EF and LF chickens were infected with ALV-J, while the negative control group showed no green fluorescence (Additional file 1C). Furthermore, the plasma samples were analysed with a p27 test for each collection, and the cell supernatant p27 test results are shown in Additional file 4. [file 13567_2021_1016_MOESM1_ESM.tif]

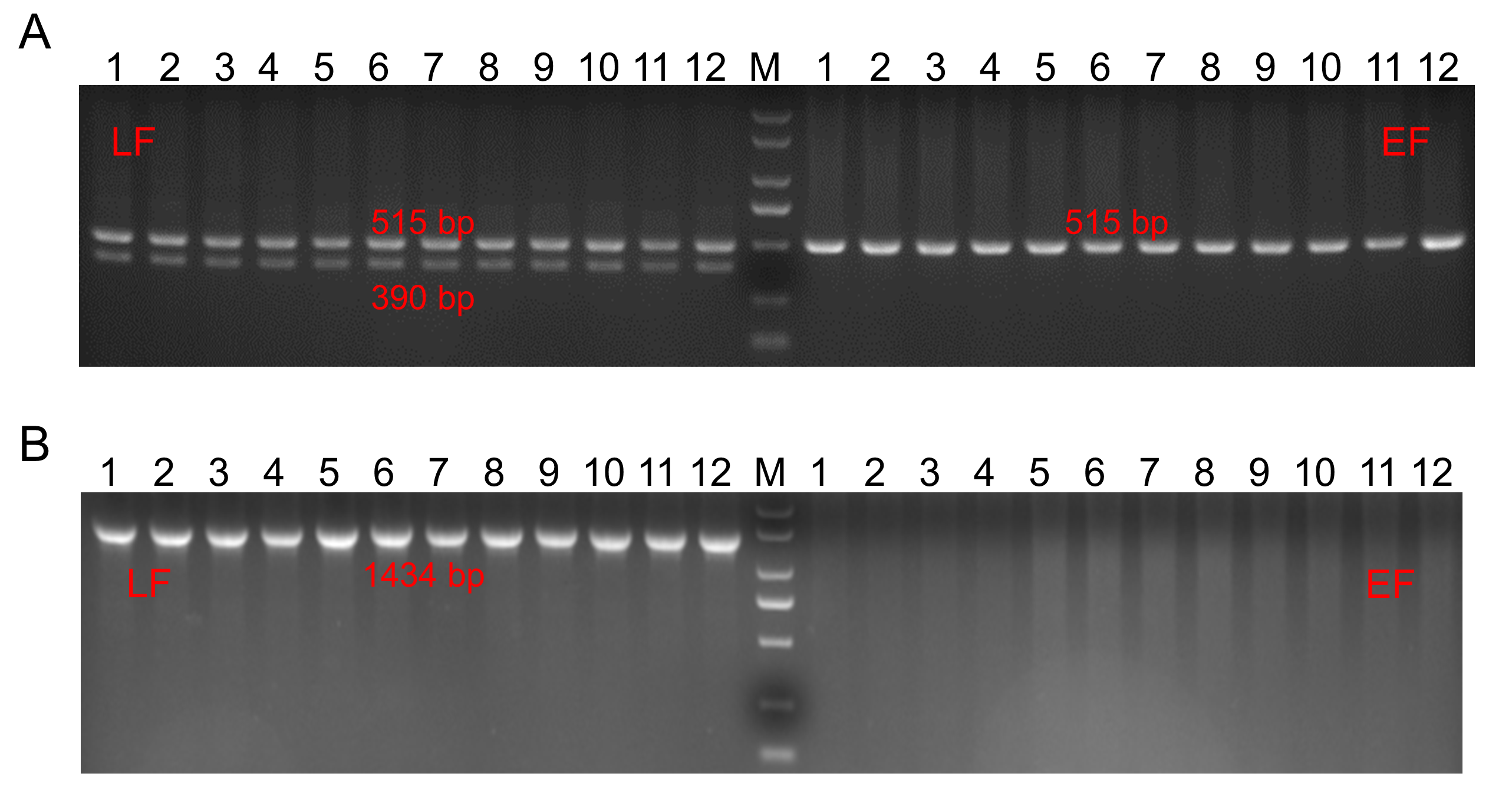

Supplement: Supplementary file 2 — Additional file 2. Detection of the ev21 and dSPEF2/dPRLR genes in sampled chickens. A The amplification results for the ev21 gene. B The amplification results for the dSPEF2/dPRLR gene. M DL2000 marker; bp base pairs; LF late feathering chicken; 1-6 LF chickens infected with ALV-J; 7-12 LF chickens not infected with ALV-J; EF early feathering chicken; 1-6 EF chickens infected with ALV-J; 7-12 EF chickens not infected with ALV-J. Note: Two target fragments (515 and 390 bp) produced with ev21 gene primers and a 1434-bp target fragment produced with dSPEF2/dPRLR gene primers were found for all LF chickens. Only one target fragment (515 bp) produced with ev21 gene primers and no target fragment produced with dSPEF2/dPRLR gene primers were found for all EF chickens (Additional file 2A and B). [file 13567_2021_1016_MOESM2_ESM.tif]
