# Supplementary material for: dPRLR causes differences in immune responses between early and late feathering chickens after ALV-J infection
Source: Vet Res. 2022 Jan 8;53:1. doi: 10.1186/s13567-021-01016-7 (PMC8742939; doi:10.1186/s13567-021-01016-7)
Supplement: Supplementary file 3 — Additional file 3. Primer information. [file 13567_2021_1016_MOESM3_ESM.docx]

| Primer | Sequence (5’–3’) | Use |
| --- | --- | --- |
| ALV-A | F: GGATGAGGTGACTAAGAAAG  R: AGAGAAAGAGGGGTGTCTAAGGAG | PCR |
| ALV-B | F: GGATGAGGTGACTAAGAAAG  R: ATGGACCAATTCTGACTCATT | PCR |
| ALV-C | F: GGATGAGGTGACTAAGAAAG  R: GAGGCCAGTACCTCCCACG | PCR |
| ALV-D | F: GGATGAGGTGACTAAGAAAG  R: ATCCATACGCACCACAGTATTCG | PCR |
| ALV-J | F: GGATGAGGTGACTAAGAAAG  R: CGAACCAAAGGTAACACACG | PCR |
| MDVMEQ | F: CCGTCTAGAAGGCGGGCACGGTAC  R: CGGAAGCTTAAACATGGGGCATAGACG | PCR |
| MDV132 | F: TGCGATGAAAGTGCTATGGAGG  R: GAGAATCCCTATGAGAAAGCGC | PCR |
| REV | F: CATACTGGAGCCAATGGTT  R: AATGTTGTAGCGAAGTACT | PCR |
| Ev21 | F: GTGGGAATGGTACTACAGAGAAGG  R: CATTTCAAGCAAGGGACTGGC  IN: ACCTGAATGAAGCTGAAGGCTTC | PCR |
| dSPEF2/dPRLR | F: CTGAGAGTGTTGTCCCAGCA  R: TGTTGAGTGCTCTTGGTTGC | PCR |
| TLR4 | F: AGGCACCTGAGCTTTTCCTC  R: TACCAACGTGAGGTTGAGCC | qRT-PCR |
| TLR7 | F: TCTGGACTTCTCTAACAACA  R: AATCTCATTCTCATTCATCATCA | qRT-PCR |
| MDA5 | F: ATTCCACAGCCGCAGATTC  R: CAAGATTGGCACAGATTTTCAGA | qRT-PCR |
| SOCS3 | F: CAGTACGATGCCCCCCTCTA  R: TCCCAGAACGAGGGGAACGA | qRT-PCR |
| VIP | F: TCCTGTCAAACGCCACTCTG  R: AGGTGGCTCAGCAGTTCATCTA | qRT-PCR |
| IRF-1 | F: GATGGACGTGTTCTCCACCT  R: TCCCACCTCTGACAAAGGAC | qRT-PCR |
| NFkB | F: GCTGCTTTGACACAGATGGA  R: CCTTTGCAAAACTGGTTGGT | qRT-PCR |
| TNFa | F: AGCAGGGCTGACACGGAT  R: TGTTGGCATAGGCTGTCCTG | qRT-PCR |
| IL-1β | F: GGTCAACATCGCCACCTACA  R: CATACGAGATGGAAACCAGCAA | qRT-PCR |
| IL-10 | F: AGCAGATCAAGGAGACGTTC  R: ATCAGCAGGTACTCCTCGAT | qRT-PCR |
| GAPDH | F: GAACATCATCCCAGCGTCCA  R: CGGCAGGTCAGGTCAACAAC | qRT-PCR |
