# Supplementary material for: dPRLR causes differences in immune responses between early and late feathering chickens after ALV-J infection
Source: Vet Res. 2022 Jan 8;53:1. doi: 10.1186/s13567-021-01016-7 (PMC8742939; doi:10.1186/s13567-021-01016-7)
Supplement: Supplementary file 4 — Additional file 4. ALV-J viremia was detected by ALV group-specific antigen (p27) ELISA. Note: LF Po LF chickens infected with ALV-J; LF Ne LF chickens not infected with ALV-J; EF Po EF chickens infected with ALV-J; EF Ne EF chickens not infected with ALV-J; W, week. The results for viremia are expressed as the S/P value. An S/P value > 0.2 indicates the presence of ALV-J viremia. [file 13567_2021_1016_MOESM4_ESM.docx]

| Chicken | S/*P* value | | | | | | | |
| --- | --- | --- | --- | --- | --- | --- | --- | --- |
|  | 1w | 2w | 3w | 4w | 5w | 6w | 7w | 8w |
| LF Po-1 | 2.360 | 2.010 | 0.185 | 0.149 | 0.310 | 0.330 | 1.440 | 2.040 |
| LF Po-2 | 2.300 | 2.410 | 0.710 | 0.720 | 0.890 | 1.130 | 1.540 | 2.100 |
| LF Po-3 | 2.520 | 2.180 | 1.670 | 1.430 | 1.760 | 1.770 | 2.100 | 2.110 |
| LF Po-4 | 1.630 | 2.040 | 0.010 | 0.017 | 0.003 | 0.039 | 0.013 | 1.190 |
| LF Po-5 | 0.760 | 2.230 | 0.007 | 0.002 | 0.059 | 0.010 | 0.006 | 0.024 |
| LF Po-6 | 2.280 | 2.450 | 0.300 | 0.400 | 0.970 | 1.600 | 2.090 | 2.150 |
| EF Po-1 | 2.350 | 2.420 | 1.850 | 1.560 | 1.840 | 1.990 | 0.004 | 2.030 |
| EF Po-2 | 2.290 | 2.250 | 1.820 | 1.590 | 1.880 | 1.910 | 2.170 | 1.830 |
| EF Po-3 | 2.570 | 2.180 | 1.860 | 1.770 | 2.020 | 1.980 | 2.080 | 1.950 |
| EF Po-4 | 2.270 | 2.480 | 0.270 | 1.660 | 1.330 | 1.580 | 1.800 | 0.053 |
| EF Po-5 | 2.180 | 2.390 | 1.930 | 1.620 | 1.950 | 1.800 | 2.020 | 1.850 |
| EF Po-6 | 2.200 | 2.520 | 1.530 | 0.230 | 1.930 | 1.960 | 2.540 | 2.080 |
| LF Ne-1 | -0.003 | 0.011 | -0.004 | 0.001 | -0.002 | -0.002 | -0.002 | -0.003 |
| LF Ne-2 | 0.003 | -0.003 | 0.004 | -0.002 | 0.000 | -0.001 | -0.003 | -0.004 |
| LF Ne-3 | -0.001 | -0.002 | -0.004 | -0.003 | -0.001 | -0.002 | 0.008 | -0.004 |
| LF Ne-4 | -0.003 | 0.012 | 0.003 | 0.000 | 0.004 | 0.000 | 0.004 | 0.000 |
| LF Ne-5 | -0.003 | 0.022 | -0.005 | 0.000 | -0.001 | -0.002 | -0.003 | 0.000 |
| LF Ne-6 | 0.040 | 0.015 | -0.003 | -0.001 | 0.001 | -0.003 | 0.014 | -0.001 |
| EF Ne-1 | 0.004 | -0.002 | 0.009 | 0.002 | 0.004 | 0.003 | 0.003 | -0.003 |
| EF Ne-2 | 0.044 | -0.001 | -0.002 | 0.020 | -0.001 | 0.003 | 0.004 | -0.003 |
| EF Ne-3 | 0.019 | 0.009 | 0.005 | 0.042 | 0.000 | -0.001 | 0.042 | 0.001 |
| EF Ne-4 | 0.005 | 0.003 | 0.003 | 0.002 | 0.006 | 0.001 | 0.003 | 0.003 |
| EF Ne-5 | 0.007 | -0.001 | 0.008 | 0.010 | 0.001 | -0.001 | 0.005 | 0.000 |
| EF Ne-6 | 0.025 | -0.001 | -0.001 | 0.004 | 0.016 | 0.008 | 0.013 | 0.004 |
